# Childhood sarcoidosis: A rare but fascinating disorder

**DOI:** 10.1186/1546-0096-6-16

**Published:** 2008-09-23

**Authors:** Avinash K Shetty, Abraham Gedalia

**Affiliations:** 1 Division of Pediatric Infectious Diseases, Wake Forest University Health Sciences and Brenner Children's Hospital, Winston-Salem, NC, USA; 2 Division of Pediatric Rheumatology, Louisiana State University Medical Center and Children's Hospital of New Orleans, LA, USA

## Abstract

Childhood sarcoidosis is a rare multisystemic granulomatous disorder of unknown etiology. In the pediatric series reported from the southeastern United States, sarcoidosis had a higher incidence among African Americans. Most reported childhood cases have occurred in patients aged 13–15 years. Macrophages bearing an increased expression of major histocompatibility class (MHC) II molecules most likely initiate the inflammatory response of sarcoidosis by presenting an unidentified antigen to CD4+ Th (helper-inducer) lymphocytes. A persistent, poorly degradable antigen driven cell-mediated immune response leads to a cytokine cascade, to granuloma formation, and eventually to fibrosis. Frequently observed immunologic features include depression of cutaneous delayed-type hypersensitivity and a heightened helper T cell type 1 (Th1) immune response at sites of disease. Circulating immune complexes, along with signs of B cell hyperactivity, may also be found. The clinical presentation can vary greatly depending upon the organs involved and age of the patient. Two distinct forms of sarcoidosis exist in children. Older children usually present with a multisystem disease similar to the adult manifestations, with frequent hilar lymphadenopathy and pulmonary infiltrations. Early-onset sarcoidosis is a unique form of the disease characterized by the triad of rash, uveitis, and arthritis in children presenting before four years of age. The diagnosis of sarcoidosis is confirmed by demonstrating a typical noncaseating granuloma on a biopsy specimen. Other granulmatous diseases should be reasonably excluded. The current therapy of choice for sarcoidosis in children with multisystem involvement is oral corticosteroids. Methotrexate given orally in low doses has been effective, safe and steroid sparing in some patients. Alternative immunosuppressive agents, such as azathioprine, cyclophosphamide, chlorambucil, and cyclosporine, have been tried in adult cases of sarcoidosis with questionable efficacy. The high toxicity profile of these agents, including an increased risk of lymphoproliferative disorders and carcinomas, has limited their use to patients with severe disease refractory to other agents. Successful steroid sparing treatment with mycophenolate mofetil was described in an adolescent with renal-limited sarcoidosis complicated by renal failure. Novel treatment strategies for sarcoidosis have been developed including the use of TNF-alpha inhibitors, such as infliximab. The long-term course and prognosis is not well established in childhood sarcoidosis, but it appears to be poorer in early-onset disease.

## Introduction

Sarcoidosis is a multisystem systemic granulomatous disease of unknown etiology that most commonly affects young adults, who frequently present with hilar lymphadenopathy, pulmonary infiltration, and ocular and cutaneous lesions [1]. Although the lung is most frequently involved, the disease can affect any organ system of the body [2]. The disease is relatively rare in the pediatric population [3,4]. Infants and children younger than 5 years usually present with the triad of skin, joint, and eye involvement, without typical lung disease. However, older children have involvement of the lungs, lymph nodes, and eyes more frequently, as seen in adult [5,6].

Despite a variety of hypotheses regarding causative agents, the cause of sarcoidosis is unknown [7]. The definitive diagnosis of sarcoidosis is made when compatible clinical findings are associated with histopathological evidence of noncaseating granulomata in affected organs and other granulomatous disorders are excluded [8]. The illness can be self-limited or chronic, and the disease characteristics vary among various populations [1]. The course and prognosis of sarcoidosis in children is different compared to adults, and may correlate with the mode of onset and the extent of the disease [1,9,10].

In this review, we discuss the epidemiology, pathogenesis, etiology, and clinical features of sarcoidosis in children, and examine the current approaches to diagnosis and treatment of this enigmatic disease.

## Epidemiology

The prevalence of sarcoidosis in the adult population ranges from 10 to 40 per 100,000 in the United States and Europe [1]. The true incidence and prevalence of childhood sarcoidosis is unknown because of the rarity of the disease and the small number of reported cases in childhood. A recent review reported that the approximate incidence of clinically recognized sarcoidosis in Danish children younger than 15 years was 0.22–0.27 per 100,000 children per year, corresponding to approximately 3 new cases in Denmark each year [4]. As in adults, many children with sarcoidosis may be asymptomatic and the disease may remain undiagnosed.

Most reported childhood cases have occurred in patients aged 13–15 years [11,12]. In a recent international registry study of childhood sarcoidosis associated with joint involvement, the mean age at onset was 10.6 years (range, 0.1–16 years) [13]. Early-onset childhood sarcoidosis (ie, with onset in the first 4 y of life) is rare but well described [5,6].

Adult studies have reported a slightly higher disease rate for women. A population-based study of incidence and survival in adults with sarcoidosis reported incidence rates of 5.9 per 100,000 person-years for men and 6.3 per 100,000 person-years for women. No clear sex predominance exists in childhood sarcoidosis. In a recent study from Denmark, the male/female gender ratio was close to one [4].

The racial distribution of sarcoidosis varies with geographic location. In the US adult population, sarcoidosis occurs in about 35.5 per 100,000 blacks and 11 per 100,000 whites. Studies in military and veteran populations showed that blacks are 10–17 times more commonly affected with sarcoidosis than are whites [1]. In the pediatric series reported from the southeastern United States, sarcoidosis had a higher incidence among African Americans [11,14]. In children aged 4 years and younger with sarcoidosis, 7–28% are African Americans, whereas in children aged 8–15 years, the percentage of African Americans increases to 72–81% [5,11,14]. Outside the United States, sarcoidosis most frequently occurs in the predominant race of the country. Thus, in Scandinavian countries where sarcoidosis is common, almost all cases occur in white people, while in Japan, most patients are Asian [1,15].

Within the US, approximately 80% of childhood cases have been reported primarily in Virginia, North Carolina, South Carolina, and Arkansas, and Louisiana, suggesting that the southeastern and south central states are an endemic area for childhood sarcoidosis [11,14,16,17].

## Genetics of sarcoidosis

Familial aggregation and the striking racial variation in sarcoidosis incidence suggest a genetic predisposition to develop sarcoidosis [18]. The strongest support for a genetic susceptibility to sarcoidosis comes from numerous reports of familial clustering of cases [19,20]. In the United States, familial clusters more frequently are observed in African Americans, with a rate of at least 19% in affected African American families as compared to 5% in white families. When sarcoidosis has been observed in twins, monozygotic twins are 2–4 times more concordant for disease than dizygotic twins [19]. The most common familial relationship is sibling pairs, followed by parent-offspring [19]. The hereditary differences in candidate genes that promote susceptibility may reside in loci that influence regulation of antigen presentation or recognition, T cell function, or the regulation of matrix deposition that favors granuloma formation and progressive fibrosis.

Genetic factors may also be important in defining the pattern of disease presentation, its severity, and prognosis. Several investigators have searched for associations with human leukocyte antigen (HLA)-related genes that may confer susceptibility to sarcoidosis [21]. A genome-wide linkage analysis performed in German families with follow-up fine mapping studies has revealed a unique candidate gene, BTNL2 in the MHC II region on chromosome 6 [22]. A follow-up study found that sarcoidosis is associated with a truncating splice site mutation in BTNL2 [23]. The BTNL2-conferred sarcoidosis risk has been noted in both Caucasians and African-Americans [24]. A genome-wide scan performed in African-American families with follow-up fine mapping studies has indicated chromosome 5 as potentially harboring candidate genes [25].

## Etiology

Despite the global occurrence of sarcoidosis, the etiology of sarcoidosis is unknown. Most experts think that sarcoidosis results from exposure of genetically susceptible hosts to specific environmental, occupational or infectious agents that trigger an exaggerated cellular immune response, leading to granuloma formation [20,26]. Despite extensive research, no defined cause has been demonstrated to account for the granulomata that characterize the disease [26-28].

Case clustering, and immunologic and clinical similarities of sarcoidosis to infectious granulomatous diseases suggests that an infectious agent may be responsible. Mycobacteria, including *Mycobacterium tuberculosis *and other atypical species have received the greatest amount of attention [29-32]. The detection of mycobacterial DNA in sarcoid lesions by polymerase chain reaction (PCR) lends support to that association [32]. A recent molecular study identified a novel candidate tissue antigen of mycobacterial origin, the Mycobacterium tuberculosis catalase-peroxidase protein (mKatG) in sarcoid granulomas as a potential target of the adaptive immune response in sarcoidosis [31].

Besides *Mycobacteria*, many different antigens are under suspicion, including other bacteria such as *Proprionibacterium acnes *[32]. However, results from different studies have varied considerably and have failed in their attempts to fulfill the Koch postulates. Therefore the role of mycobacteria and proprionibacteria in the pathogenesis of sarcoidosis remains a controversial issue [33]. Recent report by Izbicki and colleagues revealed increase incidence of sarcoidosis among fire fighters in NY City that were involved in the 2001 disaster. This suggests that exposure to world trade center "dust" may play a role in sarcoidosis etiology [34].

## Immunopathogenesis

Sarcoidosis is a chronic inflammatory disease characterized by a highly focused exaggerated immune response to an unknown antigen(s) at the target organs. The hallmarks of the disease, sarcoid granulomas, most likely are formed in response to a persistent, poorly degradable, antigenic stimulus [1]. Macrophages, bearing increased expression of major histocompatibility class (MHC) II molecules, most likely initiate the inflammatory response of sarcoidosis by presenting an unidentified antigen to CD4^+ ^Th (helper-inducer) lymphocytes. This results in proliferation and activation of the T cell [35].

The immunopathology has been best studied in bronchoalveolar lavage fluid and lung disease in which early lesions consist of an alveolitis with a high proportion of activated CD4+ Th1 cells, which may precede granuloma formation [1,36]. CD4^+ ^and CD8^+ ^T-lymphocytes, as well as a few B-lymphocytes, form a characteristic ring at granuloma periphery [37]. Most granuloma-associated lymphocytes have a Th1 phenotype, secreting cytokines, including interferon gamma, interleukin (IL) -2, IL-12, and tumor necrosis factor-alpha (TNF-α), which is likely to favor the granulomatous response at sites of disease activity [38]. Additionally, alveolar macrophages release a variety of cytokines, including TNF-alpha, IL-1, IL-6, IL-12, IL-15, and growth factors in patients with sarcoidosis and pulmonary disease [35,37]. IL-18, a monocyte/macrophage-derived cytokine has been recently identified as an IFN-gamma-inducing factor and plays an important role in the induction of Th1 response and sarcoid granuloma formation [39].

Chemokines may play an important role in the pathogenesis of sarcoidosis [35]. Chemokine receptors, CXCR3 and CXCR6 are co-expressed by Th1 cells infiltrating the lung and the granuloma of patients with sarcoidosis [40]. Recent data show that T cells expressing CCR6, CXCR3, and CXCR6 act coordinately with respective ligands and Th1 inflammatory cytokines in the alveolitic/granuloma phases of the disease [41].

The CD4+ lymphocytes, in association with other immune effector cells, such as macrophages, mast cells, and natural killer cells, perpetuate the inflammatory response by release of cytokines, monocyte chemotactic factors, macrophage migration inhibitory factor, leukocyte inhibitory factor, adhesion molecules (CD49a, CD54, CD102), and growth factors [35,36].

As a result of these various immunologic interactions, an acute and often a chronic cascade of inflammation occur. This is characterized by changes in tissue permeability, cellular influx, and local cell proliferation, resulting in a granuloma. Persistent antigenic stimulation is believed to maintain the pathogenic processes. Sarcoid granulomas either resolve or heal by fibrosis. Genetic polymorphisms may influence the clinical expression of sarcoid granuloma and disease outcome [35].

Other immunologic abnormalities observed in patients with sarcoidosis include circulating immune complexes, B-cell hyperactivity, spontaneous in situ production of immunoglobulins, and depression of cutaneous delayed-type hypersensitivity reactions [1].

## Clinical manifestations

Because sarcoidosis is a multisystem disease and affects most organs, the clinical presentation can vary greatly. In most children, the disease frequently involves the lungs, lymph nodes, eyes, skin, liver, and spleen [4,7,9]. Most of the reported cases of childhood sarcoidosis are accompanied by nonspecific constitutional symptoms, such as fever, fatigue, malaise, and weight loss, as well as symptoms from particular organs such as lungs, eyes, skin, and lymph nodes. [4,9,14,15]. The disease can be asymptomatic in children and remain undiagnosed [4].

Two distinct forms of childhood sarcoidosis appear to exist. Older children usually present with a multisystem disease similar to the adult manifestation with frequent lymphadenopathy and pulmonary involvement, as well as generalized signs and symptoms, such as fever, malaise, and weight loss [4,13-15]. In contrast, early-onset childhood sarcoidosis is a unique form of the disease characterized by the triad of rash, uveitis, and arthritis in children who are younger than 5 years [5,6,10].

### Pulmonary manifestations

**Figure 1:**
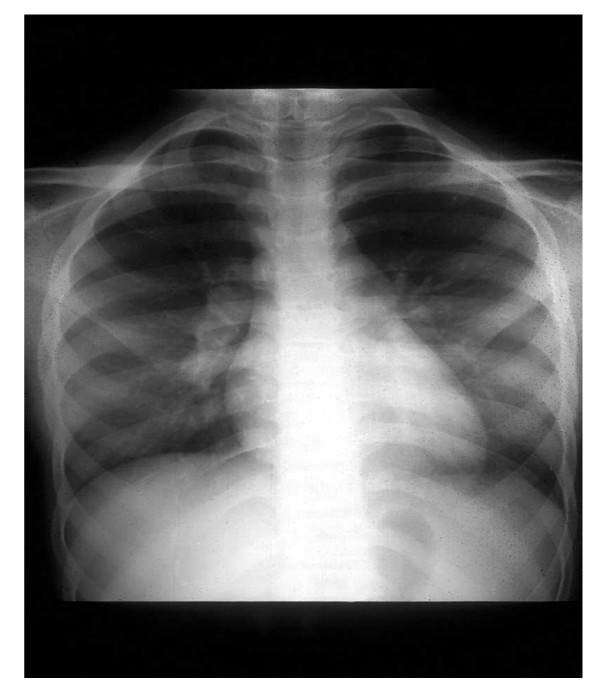
Chest radiograph in a 10-year-old female with sarcoidosis showing hilar adenopathy.

**Figure 2:**
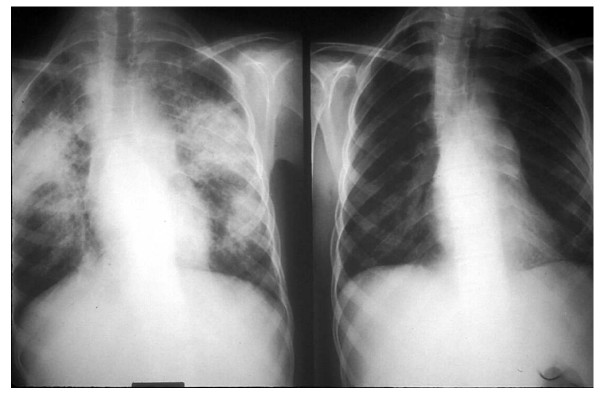
**Pulmonary infiltrates of sarcoidosis: Chest radiograph in a 12-year-old female with sarcoidosis showing patchy, diffuse alveolar infiltrates involving both lungs (Left). **A repeated study 6 months later with significant improvement (Right).

The lung is the organ most commonly involved in sarcoidosis [1,2,7]. Pulmonary symptoms are usually mild and often consist of a dry hacking cough, with or without mild to moderate dyspnea, and occasionally chest pain [15]. Physical examination is often unremarkable, but crackles, ronchi, wheezing, or diminished breath sounds may be noted. Bilateral hilar lymphadenopathy with or without parenchymal involvement is the most common radiographic finding [4,42] (Figures 1). Typically, the hilar lymphadenopathy is symmetrical, although in rare instances it may appear unilateral. Parenchymal involvement is usually an interstitial pattern, although nodular, alveolar, and fibrotic patterns also are described [42] (Figure 2).

In a recent Danish study, chest radiographs were normal (stage 0) in 10%; 71% had isolated bilateral hilar lymphadenopathy (stage I), 8.3% had bilateral hilar adenopathy with pulmonary infiltrates (stage II) and 8.3% had parenchymal infiltrates without hilar adenopathy (stage III). None of the 48 reported patients had evidence of fibrosis (stage IV) [4].

Nearly half of all children with sarcoidosis demonstrate restrictive lung disease on static and dynamic pulmonary function tests [4,7,11,43]. These changes are nonspecific and believed to be secondary to the early alveolitis progressing to fibrosis. Obstructive ventilatory defects secondary to intrabronchial sarcoid granuloma or hilar/mediastinal lymph node compression of the airways or bronchiectasis may be occasionally seen [11,15,43].

### Reticuloendothelial System

The most common physical sign of reticuloendothelial involvement is peripheral lymph node enlargement noted in 40% to 70% of cases [4,11,14], which is also the most accessible site for diagnostic biopsy [15]. Lymph nodes are typically firm, non-tender, and freely movable. Although hepatosplenomegaly may occur in up to 43% of patients with childhood sarcoidosis [11], clinical manifestations are not as apparent [9].

### Ocular manifestations

Ocular involvement is common in childhood sarcoidosis. Visual symptoms such as eye pain, blurry vision, photophobia, and redness may be present in 29% of the patients [4,44]. Anterior segment disease consisting of uveitis or iritis is the most common manifestation occurring in 24% to 58% of the children with sarcoidosis [4,6,9,11,13,15]. Uveitis of sarcoidosis is characterized by firmly-edged keratic precipitates, most commonly develop in the lower part of the cornea and also seen in the limbus; iris nodules; and focal synechiae related to nodule formation. However, the majority of the synechiae are caused by adhesions between iris and lens due to inflammation [11]. Chorioidal granuloma and peripheral multifocalchorioiditis are very specific for ocular sarcoidosis. Conjunctival granulomas are the second most common ocular manifestation in sarcoidosis and may appear as tiny, translucent, pale yellow nodules [44]. Other ocular lesions can include keratitis, retinitis, glaucoma, and involvement of the eyelids and lacrimal glands [44]. Ophthalmological slit lamp examination is mandatory in the evaluation of childhood sarcoidosis [9]. If left untreated, serious complications including blindness can occur [6,10,13].

### Skin manifestations

**Figure 3:**
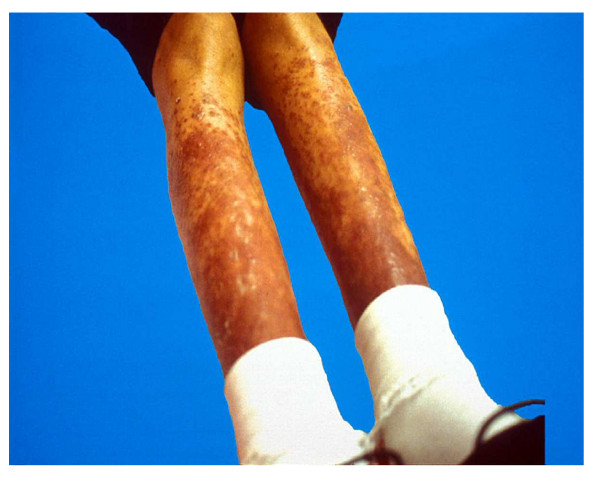
Skin rash in a 12-year-old female with sarcoidosis: Both lower extremities showing elevated, diffuse purplish-colored nodular and scaly rash.

An erythematous rash is commonly noted in childhood sarcoidosis and occurs in 77% of young children and 24–40% in older children [4,5,11,14] (Figure 3). The most frequent cutaneous eruptions include soft, red to yellowish brown, or violaceous, flat-topped papules, found most frequently on the face [3]. In children, macular lesions with scarring and ichthyosiform cutaneous manifestations are frequently encountered [45]. Larger, violaceous, plaque-like lesions may be found on the trunk, extremities, and buttocks. In a recent study, erythema nodosum was noted in 31% of the children [4]. Other skin lesions of sarcoidosis include nodules, hyperpigmented or hypopigmented lesions, ulcers, and subcutaneous tumors [3,4].

### Musculosketetal involvement

Musculosketelal features of sarcoidosis include joint effusions, joint pain, and, rarely, osseous involvement. Arthritis has been reported in 15% to 58% of children with sarcoidosis [4,5,11]. The arthritis of childhood sarcoidosis is characterized by boggy tenosynovitis with relatively painless effusion and good range of movement with often no overlying erythema of the skin [46]. Multiple joints of both the upper and lower extremities are involved. Radiographic evidence of joint erosions or juxtaarticular osteoporosis is usually absent. Sarcoid arthritis can be confused with juvenile rheumatoid arthritis (JRA) in young children. Bone involvement is rarely noted in children with sarcoidosis [15]. Symptomatic muscle involvement is unusual both in adults and children.

### Renal involvement

Renal involvement occurs infrequently in children with sarcoidosis [11,15,47]. This is often related to hypercalcemia and hypercalciuria or less frequently to infiltration of renal tissues with sarcoid granulomas [9,11,47]. The clinical manifestations in reported cases of renal granulomatous sarcoidosis include proteinuria, leucocyturia, hematuria, concentration defect, hypertension, membranous nephropathy, interstitial nephritis, and renal failure [48,49]. In a previous review, decreased creatinine clearance was found in more than 60% of children with sarcoidosis, although other evidence of renal dysfunction, such as abnormal urinalysis results and elevated blood urea nitrogen and serum creatinine values, were found in less than 40% of children [11].

Derangement in calcium metabolism manifesting as hypercalcemia and/or hypercalciuria, occurs in up to 30% of children, and can be associated with nephrocalcinosis and nephrolithiasis [4,11,48]. In vitro experiments of cultured alveolar macrophages from patients with sarcoidosis and of homogenized sarcoid lymph node tissue have demonstrated that the sarcoid macrophage is able to synthesize 1,25-dihydroxyvitamin D via 25-hydroxyvitamin D3-1-alpha hydroxylating activity. The excess circulating 1,25-dihydroxyvitamin D produced extrarenally by the granulomas causes increased intestinal absorption of calcium, enhanced bone resorption, and resultant hypercalciuria, with or without hypercalcemia [50].

### Other organ systems

Parotid gland enlargement is a frequent finding in children with sarcoidosis, especially the early onset type [6]. Sarcoidosis involving the heart has been well-documented in adults but rarely in children [6,10]. Clinical manifestations of sarcoid cardiac disease may be varied and include heart block, cardiomyopathy, or ventricular arrhythmias. Vasculitis associated with sarcoidosis is unusual but has been reported [3]. The majority of reports cite involvement of small- to medium-size vessels contiguous to diseased tissue, although large-vessel vasculitis associated with abdominal aortic aneurysm has been described [3,51]. Involvement of the central nervous system disease, although common in adults, are rare in children [52].

## Early-onset childhood sarcoidosis and Blau syndrome

Early-onset childhood sarcoidosis, i.e., with onset in the first 5 years of life differs from sarcoidosis in older children and adults and often poses a diagnostic challenge to the clinician [5,6,10,15]. Typically presenting in the first year of life [10], patients with early-onset disease exhibit unique clinical features characterized by the triad of arthritis, rash, and uveitis. [5,6] Hilar lymphadenopathy, the leading feature of late-onset forms [15], is rare in early-onset sarcoidosis [5]. Uveitis, which occurs in about in more than half the children with early-onset sarcodiosis, is relatively less common in patients with later onset [6].

In 1985, Edward Blau described families with autosomal dominant granulomatous disease demonstrating the classic triad of arthritis, dermatitis, and uveitis, an identical phenotype to early-onset sarcoidosis [53]. Recent data suggests that early-onset sarcoidosis and Blau syndrome likely represent the same disease since both entities share genetic mutations in the NOD2 (nucleotide binding oligomerization domain 2), also referred to as capsule recruitment domain family member 15 (*CARD15*) in 50–90% of cases [54]. This finding has led some experts to propose the term "pediatric granulomatous arthritis" to describe both disorders [55]. Although clinical similarities between Blau syndrome and sarcoidosis suggest genetic homogeneity between them, a study by Rybicki and colleagues [56] found no linkage of sarcoidosis to the Blau syndrome locus. Therefore, Blau gene has no major effect on sarcoidosis susceptibility.

Early-onset sarcoidosis may be overlooked due to its similarity to systemic-onset JRA [45]. Both entities may be associated with systemic manifestations such as fever, weight loss, and fatigue. Skin changes, however, may help to distinguish between the two diseases at the onset. The rash of JRA is pink, evanescent, and macular, whereas the rash of sarcoidosis is classically a papule or a plaque with scaling [5]. Arthritis of sarcoidosis is characterized by painless boggy effusions of the synovium without limitation of range of movement [5,46]. However, painful, destructive polyarthritis with functional impairment indistinguishable from that associated with JRA has been described in early-onset sarcoidosis [6].

## Diagnosis

There is no laboratory test diagnostic of sarcoidosis. Laboratory evaluation may reveal elevated erythrocyte sedimentation rate (ESR) or other acute phase reactants. Anemia, leukopenia, and eosinophilia are commonly seen on blood counts [4]. Immunological abnormalities include hypergammaglobulinemia and impaired delayed hypersensitivity on skin testing [1]. Hypercalcemia and/or hypercalciuria may be found [9,11,15,47]. The serum level of angiotensin-converting enzyme (ACE) is elevated in over 50% of children with late-onset sarcoidosis [4,57,58], but the test is not specific for sarcoidosis, and many other disorders may be similarly associated with increased serum ACE activity [3].

The source of serum ACE in sarcoidosis is believed to be epitheloid cells in granulomata. ACE serum levels have been shown to be useful in diagnosing sarcoidosis and following disease activity and the effect of therapy in older children with sarcoidosis [57,58]. Reference values for serum ACE is age-dependent and healthy children have ACE levels that are 40–50% higher than in adults [58].

Chest radiograph is very useful and may reveal bilateral hilar adenopathy [4,42]. Because alveolitis precedes granuloma formation in the lungs and is the earliest sign of activity in pulmonary sarcoidosis, bronchoalveolar lavage (BAL) performed through a flexible fiberoptic bronchoscope has been used to assess that activity. [1,2] BAL typically demonstrates an increased number of lymphocytes, most of which are activated helper-inducer T lymphocytes, which can cause the CD4/CD8 ratio to be high [1,2]. However, in children BAL lymphocytosis does not correlate with disease activity, treatment response or prognosis [59]. Therefore, serial BAL is not routinely recommended in pulmonary sarcoidosis in children [9]. High-resolution chest computed tomography (CT) can be helpful in delineating the extent of parenchymal disease and hilar adenopathy. Although fibrotic lung disease is a rare consequence of childhood sarcoidosis, follow-up with pulmonary function tests (PFTs) is useful in detecting it. Other studies such as gallium-scan, have not proven to have clear prognostic benefits [3]. Another emerging tool that is been used mainly in detecting metastatic cancer is positron emession tomography (PET scan). A recent study determined that PET scan does not allow differentiation of interstitial pulmonary fibrosis (IPF) from a non-IPF pulmonary diseases including sarcoidosis [60].

**Figure 4:**
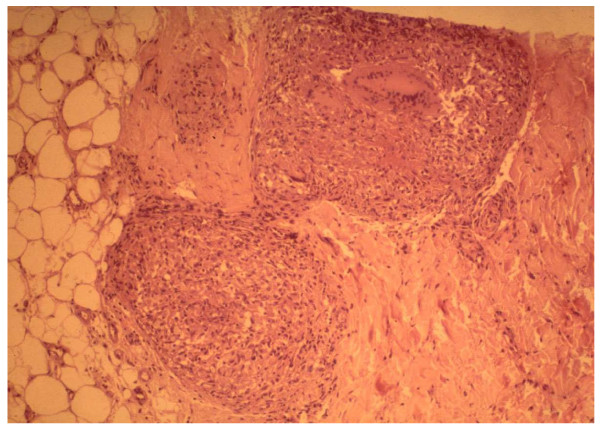
Pathology of sarcoidosis: Skin biopsy specimen from the rash in figure 3, revealing intradermal well-circumscribed, noncaseating epitheloid granulomata with typical multinucleated giant cells.

The diagnosis of sarcoidosis is confirmed by demonstrating a typical noncaseating epithelioid cell granuloma on a biopsy specimen [61] (Figure 4). To further support the diagnosis of sarcoidosis, infectious granulomatous conditions (such as histoplasmosis, blastomycosis, and tuberculosis) must be excluded by special stains and cultures [1,3]. The preferred non-invasive sites of biopsy in children include peripheral lymph node, skin lesion, enlarged salivary glands or lacrimal gland and conjunctival nodule. Other potential sites for tissue biopsy include the bone, bone marrow, liver, and lungs. The histopathological findings noted in sarcoidosis have been observed in a number of other disorders, such as tuberculosis, leprosy, Sjogren syndrome, Behcet disease and berylliosis [1,9,61].

Occasionally, sarcoidosis may present initially as fever of unknown origin (FUO) and localized bone pains, mimicking a number of infectious, inflammatory, and neoplastic processes. In such cases, magnetic resonance imaging (MRI) may reveal multifocal small nodular lesions within the marrow and help localize lesions for diagnostic exploration [62].

## Differential diagnosis

The differential diagnosis depends largely on the clinical presentation of sarcoidosis [15]. It is critical to exclude granulomatous pulmonary infections, especially those caused by mycobacteria and fungi [3]. Exclude neoplastic diseases, such as lymphoma, in cases with hilar adenopathy. Hypercalcemia in sarcoidosis may mimic metabolic disorders, such as primary hyperparathyroidism [3,15]. Early-onset sarcoidosis is often misdiagnosed as systemic-onset JRA [45]. Rarely, severe symptomatic bone marrow involvement may mimic a number of infectious and neoplastic disorders [62].

## Treatment

The current therapy of choice for childhood sarcoidosis with multisystem involvement is corticosteroids [4,7,9,15,63]. Dose and duration of therapy must often be individualized. Oral prednisone or prednisolone is usually initiated at 1–2 mg/kg/d for 4–8 weeks as induction treatment [15,63]. This treatment is continued until the clinical manifestations of the disease resolve or show significant improvement. The steroid dosage is slowly tapered in treatment responders over a period of 2–3 months to an appropriate maintenance dose (i.e., the lowest dose that controls activity of the disease, typically 10–15 mg per day as a once daily regimen) [63]. Depending on which organs are involved and on the activity of the disease, maintenance treatment is usually required for at least 6 months for most age groups and then tapered if possible [64]. Asymptomatic patients with isolated bilateral hilar adenopathy may not need systemic steroid therapy [64].

Some patients may relapse, either during steroid taper or after discontinuation of the drug. In such cases, steroid treatment should be restarted with a dosage similar to that used in the induction treatment [65]. Few patients are steroid-dependent and need long-term treatment with relatively high doses to achieve satisfactory responses. In such cases, serious complications due to chronic corticosteroid therapy, such as growth failure and bone disease, may occur [3]. Moreover, a subset of patients with persistent active or progressive sarcoidosis may be unresponsive to corticosteroids; therefore, alternative agents are needed [3,56,60,61].

Other immunosuppressive agents, especially low-dose methotrexate (MTX) have been used to treat adult patients with sarcoidosis who have steroid-resistant disease or in those with unacceptable adverse effects from glucocorticoids with good success [64]. In 1997, Gedalia and colleagues [65] described their experience with 6 months of low-dose oral MTX following a strict protocol in 7 children with biopsy-proven sarcoidosis. Corticosteroids were used for the first 6 weeks only in 6 of 7 cases. They were treated with low-dose MTX for 1 year, and the clinical response was scored using a composite of the various symptoms encountered. MTX therapy resulted in improvement in clinical symptom score and decreased mean daily steroid dosage. Additionally, parallel decreases in the ESR and the mean serum ACE concentrations were observed. No adverse effects occurred in these patients, all of whom received folate supplementation. In this study, MTX administered orally in low doses in childhood sarcoidosis was effective, safe, and had steroid-sparing properties [65]. However, because of significant concerns regarding the risk of long-term adverse effects of MTX therapy and lack of a randomized controlled trial, some experts have questioned the therapeutic index of MTX for corticosteroid-resistant sarcoidosis in children. Liver biopsy performed after a cumulative MTX dose of 1–1.5 g has been proposed to monitor liver toxicity. [3] MTX toxicity can be minimized by the use of folic or folinic acid. More recently a randomized trial of MTX in 24 adult patients with sarcoidosis by Baughman and colleagues had shown that MTX has steroid sparing property [66].

Alternative immunosuppressive agents, such as azathioprine, cyclophosphamide, chlorambucil, and cyclosporine, have been tried in adult cases of sarcoidosis with questionable efficacy [67]. The high toxicity profile of these agents, including an increased risk of lymphoproliferative disorders and carcinomas, has limited their use to patients with severe disease refractory to other agents. Successful steroid-sparing treatment with mycophenolate mofetil was described in an adolescent with renal-limited sarcoidosis complicated by renal failure [67]. Novel treatment strategies for sarcoidosis have been developed including the use of TNF-alpha inhibitors, such as infliximab [67]. Anecdotal case reports about the use of infliximab in renal sarcoidosis exist in the pediatric literature [68].

## Prognosis

The prognosis and natural history of sarcoidosis in children is unclear because of the rarity of the disease and the small number of reported series. [4,11,69,70] However, the overall prognosis of childhood sarcoidosis is reported as good compared with the prognosis for adults, with most children experiencing considerable improvement in clinical manifestations, chest radiograph findings, and pulmonary function test results [11].

In one series, Marcille et al [70] performed a follow-up study on 19 patients in whom sarcoidosis had been diagnosed in childhood. The mean follow-up period was 21 years (range 8–35 y). Seven patients (37%) had persistent abnormalities on chest radiograph, 68% had impaired lung function, and 63% had abnormal findings on echocardiography [70]. In another series from the University of North Carolina, most patients improved; however, 40% still were symptomatic and 35% had physical abnormalities after an average follow-up of 5 years [11].

Sarcoidosis in very young children with involvement of the eyes, joints and skin have a guarded prognosis with the likelihood of a chronic progressive course [5,6]; 80–100% of these children develop residua of uveitis, polyarthritis, and other organ involvement [6]. Another case series comprising 6 patients with early-onset sarcoidosis and a long follow-up with a mean of 14 years (range 0–23 y) reported on the severe outcome of the disease, including blindness (4 patients), growth retardation (3 patients), cardiac involvement (2 patients), renal failure (1 patient), and even death (1 patient) [10]. Progressive ocular disease may produce severe disability with secondary glaucoma resulting in blindness. Currently, longitudinal clinical assessment focusing on the severity of the disease in affected organs remains the best approach to prognosis. Large follow-up studies are needed to better understand long-term prognosis in childhood sarcoidosis.

## Competing interests

The authors declare that they have no competing interests.

## Consent

The parents signed a consent and gave permission to use the photos in the manuscript.
